# Bidirectional-nonlinear threshold switching behaviors and thermally robust stability of ZnTe selectors by nitrogen annealing

**DOI:** 10.1038/s41598-020-73407-3

**Published:** 2020-10-01

**Authors:** Gabriel Jang, Mihyun Park, Da Seul Hyeon, WooJong Kim, JungYup Yang, JinPyo Hong

**Affiliations:** 1 Novel Functional Materials and Device Laboratory, Research Institute of Natural Science, Department of Physics, Hanyang University, Seoul, 04763 Republic of Korea; 2 Division of Nano-Scale Semiconductor Engineering, Hanyang University, Seoul, 04763 Republic of Korea; 3 Department of Physics, Kunsan National University, Gunsan, 54150 Republic of Korea

**Keywords:** Materials for devices, Electronic devices

## Abstract

Three-dimensional stackable memory frames involving the integration of two-terminal scalable crossbar arrays are expected to meet the demand for high-density memory storage, fast switching speed, and ultra-low power operation. However, two-terminal crossbar arrays introduce an unintended sneak path, which inevitably requires bidirectional nonlinear selectors. In this study, the advanced threshold switching (TS) features of ZnTe chalcogenide material-based selectors provide bidirectional threshold switching behavior, nonlinearity of 10^4^, switching speed of less than 100 ns, and switching endurance of more than 10^7^. In addition, thermally robust ZnTe selectors (up to 400 ℃) can be obtained through the use of nitrogen-annealing treatment. This process can prevent possible phase separation phenomena observed in generic chalcogenide materials during thermal annealing which occurs even at a low temperature of 250 ℃. The possible characteristics of the electrically and thermally advanced TS nature are described by diverse structural and electrical analyses through the Poole–Frankel conduction model.

## Introduction

Two-terminal scalable crossbar array frames have attracted considerable interest as promising options for realizing three-dimensional (3D) stackable memories for use in future nonvolatile memories, such as resistive random access memory (ReRAM) and phase change memory^1–5^. Crossbar arrays are also expected to enable the effective fabrication of ideal 4F^2^ cell sizes, thereby facilitating the ongoing miniaturization of data memory storage, fast switching speed, ultralow power operation, and considerable retention characteristics. However, the widespread use of crossbar array configurations is inherently hindered by undesirable sneak-path problems arising from unintended leakage currents around neighboring unselected cells. As such, the use of suitable selectors including bidirectional nonlinear features are necessary at each cross-point node to operate the selected memory cell.

To date, numerous reports have been published on the successful development of various selectors, such as pnp junction diodes^6^, tunneling barrier^7,8^, insulator–metal transition^9^, and ovonic threshold switching (OTS)^10,11^. Of these, the OTS-based selector utilizes transient threshold switching (TS) behavior above a critical electric field level and shows promise for highly advanced nonlinearity and prominent endurance/retention features^12^, which can possibly facilitate the realization of 3D high-density crossbar arrays. The OTS-based selectors have mainly employed well-known chalcogenide materials, such as Ge_2_Sb_2_Te_5_ (GST) ternary chalcogenide doped with As, Zn, and N, due to its outstanding electrical performance and reliability characteristics^13–16^. However, the OTS-based selectors exhibit extremely large threshold voltages, which severely limit the device performances. Additionally, it requires materials with complex compositions, including arsenic as a dopant. Thus, the choice of a suitable binary OTS material-based selector without any toxic dopants or costly material is of great interest in solving the aforementioned problems for numerous applications. Among the materials considered recently, the possibility of achieving the desirable electrical performance through the use of a ZnTe binary material as the main active material of the selectors has been promising^17,18^. However, several hurdles remain due to the rapid electrical/thermal degradation that occurs during thermal annealing at temperatures above 250 ℃. We expect that this issue is likely associated with a rapid decomposition or phase separation of OTS material by thermal annealing. Thus, because thermal annealing is a generic approach requiring a high temperature of approximately 400 ℃ at the back end of the line, the manipulation of thermal stability along with achieving the desired electric characteristics is also a key challenge for future 3D stacked crossbar array semiconductor processing^19^.

In this paper, we report the TS behaviors and electrical responses of as-grown and annealed ZnTe selectors in vacuum and nitrogen ambient states. Systematic structural and electrical analyses of ZnTe thin films are done by X-ray diffraction (XRD), X-ray photoemission spectroscopy (XPS), and DC/pulse electrical characteristics to aid interpretation of the experimental findings using the Poole–Frankel conduction model. Particularly, the incorporation of a suitable nitrogen post annealing approach verifies the thermally robust electrical responses of ZnTe selectors even at a high annealing temperature of 400 ℃, which suggests the efficient protection of annealing-dependent phase separation phenomena of ZnTe active layer.

## Result and discussion

### Structural and electrical characteristics of ZnTe selector

**Figure 1:**
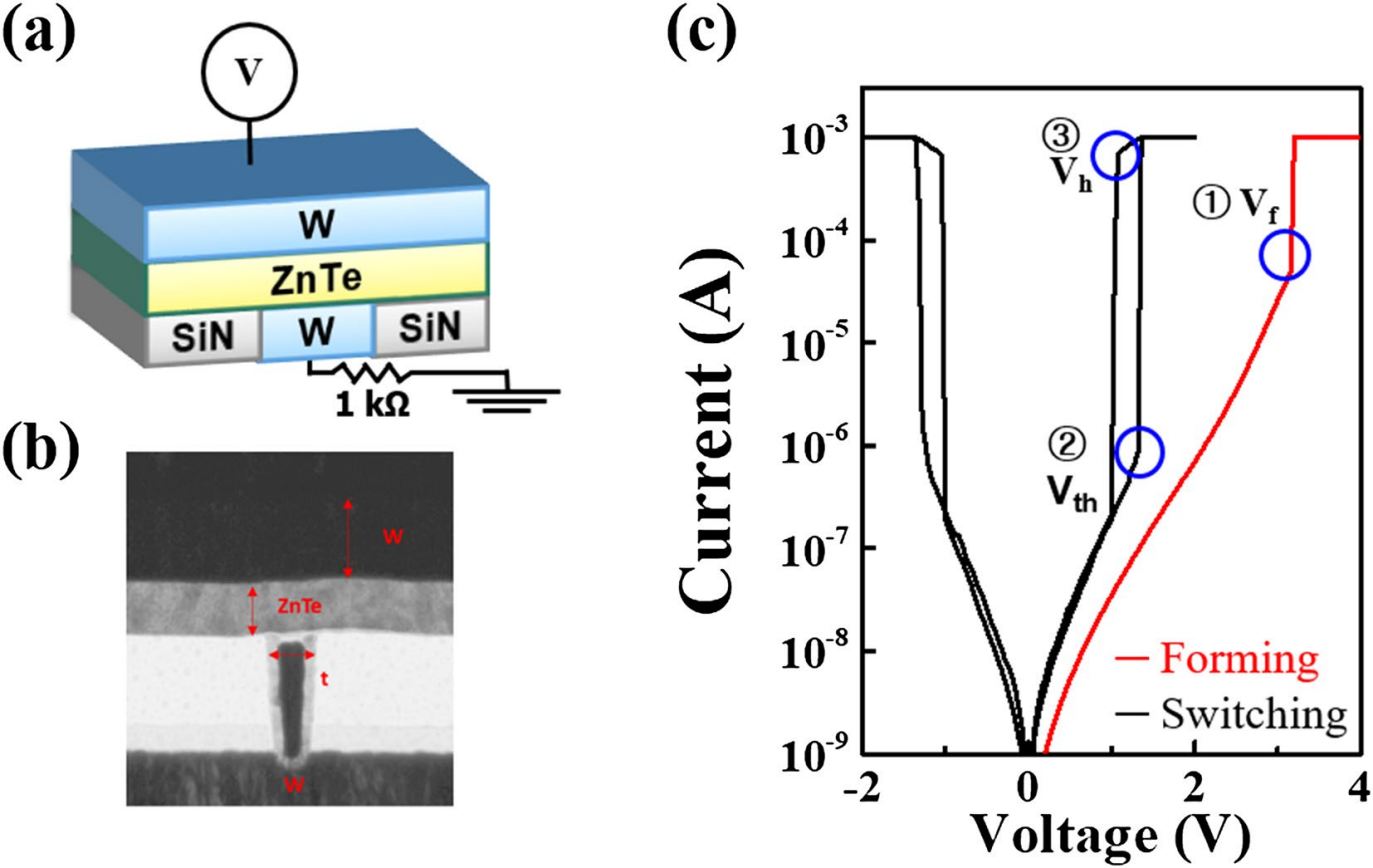
(**a**) Schematic and (**b**) cross-sectional TEM image of the W/ZnTe/W selector integrated with a serially connected resistor to avoid the overshoot during the measurements. (**c**) Representative I–V plot of the ZnTe selector, clearly revealing the forming voltage (V_f_) and bidirectional TS behavior. Over a certain voltage, namely, threshold voltage, the resistance of the TS decreases sharply. The low resistance state is maintained as long as a given voltage is above the holding voltage. (**d**) Comparison of TS curves under voltage (red line) and current (blue line) sweep modes. The voltage sweep shows the abrupt current increase/decrease at V_th_/V_h_, respectively, while the current sweep reveals the S-shape curve with a negative differential resistance event.

Figure 1a shows the schematic cross-sectional transmission electron microscope (TEM) images and representative I–V response of W/ZnTe/W selectors. The ZnTe film has a thickness of 40 nm and is sandwiched by both top and bottom W electrodes, as illustrated in Fig. 1a. The TEM image of Fig. 1b indicates the uniformly distributed growth of the ZnTe film. The representative I–V curve for as-grown ZnTe selector (Fig. 1c) reveals typical bidirectional TS behavior (black lines) after the forming process (red line) under voltage sweep modes: an initial state exhibits a high resistance, but then the resistance decreases abruptly at a forming voltage (V_f_); this process is nonreversible. After forming, the resistance decreases gradually with bias voltage sweeping until reaching the threshold voltage (V_th_) followed immediately by a sharp decrease in resistance above V_th_, clearly verifying the representative TS behavior. A similar trend also occurs in the negative region. This volatile low-resistance state is maintained as long as the applied voltage is higher than the holding voltage (V_h_). If not, it then switches to a high resistance state. Comparisons of TS curves under voltage and current sweep modes demonstrate the characteristic sharp current increases and decreases at V_th_ and V_h_ voltages, respectively, whereas the current sweep reveals the representative S-shaped curve with a negative differential resistance event (Fig. S1 in the Supplementary Information). In this study, an additional resistor in series is also employed to avoid possible overshoot problems during electrical analyses. Figure S2, in the Supplementary Information, plots the representative I–V responses of ZnTe selectors under a serially connected external resistance ranging from 1 to 10 kΩ. As shown on Fig. S2, there is no significant variation in operation voltage of V_th_ and V_h_, except for a slight variation in off- and on-current levels, regardless of the magnitudes of additional resistors.

**Figure 2:**
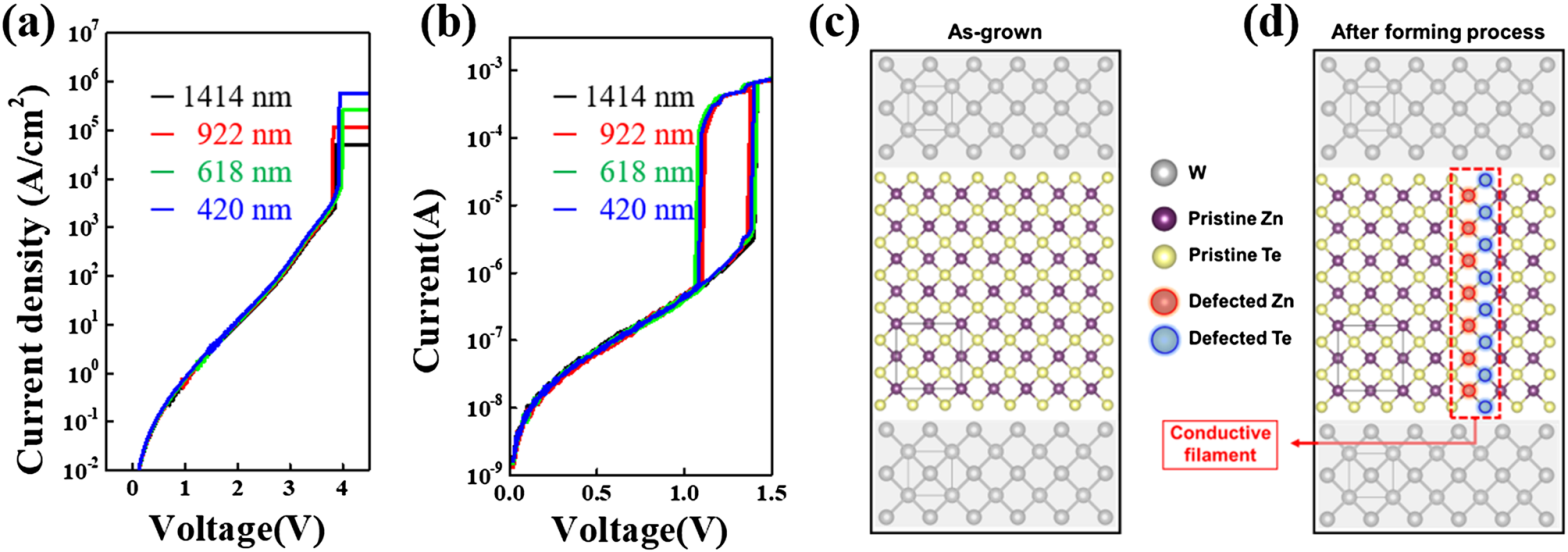
Cell-size-dependent electric features of the ZnTe selector. (**a**) Current density–voltage (I–V) and (**b**) Current–voltage (I–V) curves for the ZnTe selector before and after forming, respectively. Both characteristics clearly confirm cell-size-independent features, where the cell size is defined by the size of the bottom W electrode. Possible models of (**c**) resistive and (**d**) conductive state behaviors before and after forming, respectively. The resistive state features before forming may represent the uniform flow of initial off currents through the whole ZnTe layer between electrodes, whereas the conductive behavior after forming may reflect the presence of dominant conduction through the locally created filament paths by the forming process, which implies indirect evidence of cell-size independence.

To gain an insight into the difference in current characteristics before and after forming, which were derived from the previous work^17,18^, the cell size-dependent current density–voltage (J–V) and I–V responses for the ZnTe selector were recorded as shown in Fig. 2a,b. The cell size is defined by the size of the bottom W electrode, which is in the range of 420–1414 nm. As plotted in Fig. 2a, the initial current densities in the off-current regime before forming clearly reveal non-dependency on cell size features under identical compliance currents. Such a result in an off-current regime before forming suggests uniform current flow across the whole ZnTe layer. After forming, both off and on currents are also independent to the cell size, as seen in Fig. 2b. This may suggest the presence of the local conductive filaments (CFs) inside the ZnTe layer generated by forming^20^: that is, after forming, the local CFs represent the dominant conductance in the ZnTe active layer, whereas current flow in the other region is negligible. Figure 2c,d show the possible models for the ZnTe layer matrix before and after forming, respectively. It can be seen that the initially uniform ZnTe layer has zinc-blended crystal structures and maintains a high resistance state before forming. However, after forming, the application of a high electric field serves to break the pristine crystal state, which has a more significant impact on the generation of localized charge defects (filaments) inside the ZnTe layer, thereby contributing to the TS characteristics observed in previous work^17,18^.

**Figure 3:**
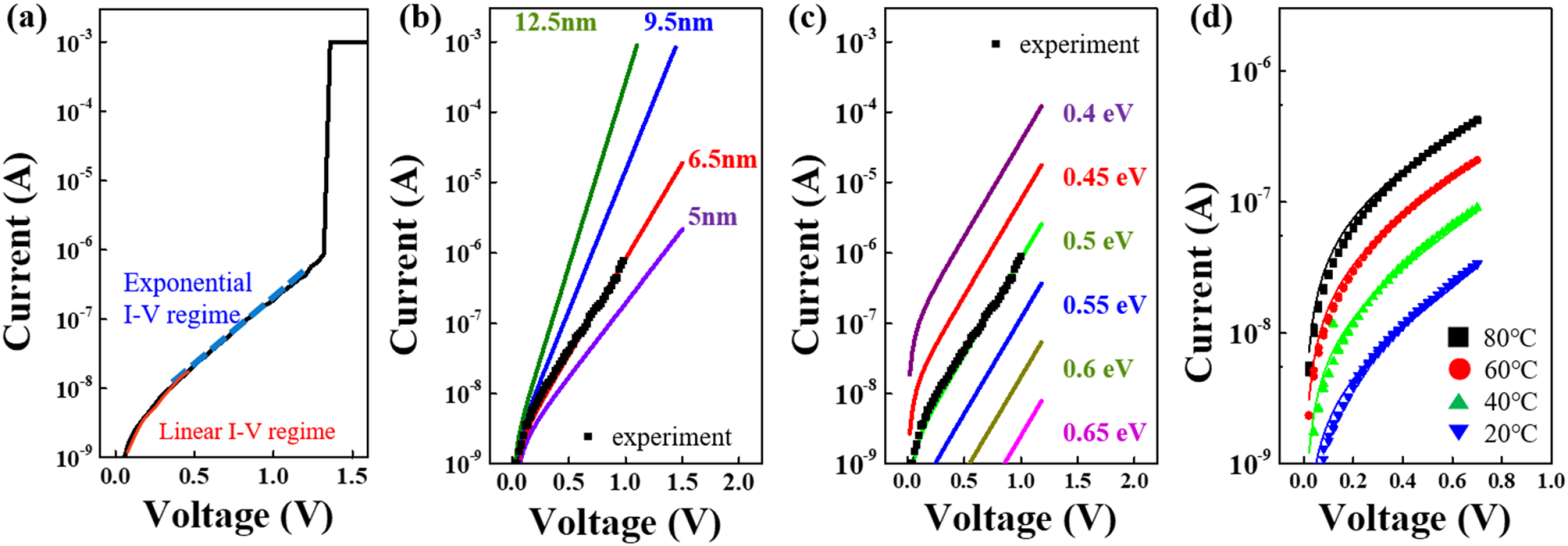
Conduction nature of Off-current regime. (**a**) I–V plot of Off-current regime, clearly demonstrating linear (red) and exponential (blue) behaviors at a certain regime boundary. Off-current mechanism of OTS materials including ZnTe is well-described by the thermal assisted hopping between dense charge traps on the basis of Poole-Frankel conduction. Analytically simulated results for (**b**) defect-to-defect distance (Δz) and (**c**) E_C_–E_F_ levels for the ZnTe selector, providing the values of Δz = 6.5 nm and E_C_–E_F_ = 0.5 eV. (**d**) Temperature-dependent Off-current behaviors, where these curves have utilized the parameters determined by (**b**) and (**c**), except temperatures causing a variation in the current levels.

To further clarify the above conduction nature before and after forming, the Poole–Frankel conduction model frequently used in previous reports is adopted for simulation, based on the observed I–V responses. This model well-describes the off-current conduction of OTS materials through thermally assisted hopping between the dense and localized charge traps^21,22^. An external electric field permits electrons to transfer by lowering the energy barrier between adjacent traps: that is, carriers can be trapped and de-trapped at defect sites under electrical stress. Below is the quantitative amount of current that flows in a subthreshold regime.

1 $${{\varvec{I}}}_{{\varvec{o}}{\varvec{f}}{\varvec{f}}}=2{\varvec{q}}{\varvec{A}}{{\varvec{N}}}_{{\varvec{T}},{\varvec{t}}{\varvec{o}}{\varvec{t}} }\frac{\Delta {\varvec{z}}}{{{\varvec{\tau}}}_{0}}\mathbf{exp}\left(-\frac{{{\varvec{E}}}_{{\varvec{C}}}-{{\varvec{E}}}_{{\varvec{F}}}}{{\varvec{k}}{\varvec{T}}}\right)\mathbf{sinh}\left(\frac{{\varvec{q}}{{\varvec{V}}}_{{\varvec{a}}}}{{\varvec{k}}{\varvec{T}}}\frac{\Delta {\varvec{z}}}{2{{\varvec{u}}}_{{\varvec{a}}}}\right)$$

where q is element charge, *A* is the region causing switching, N_T_,_tot_ is the concentration of trap, τ_0_ is the time-to-escape of traps, Δz is the trap-to-trap (or defect-to-defect) distance, kT is the thermal energy, and u_a_ is the thickness of the ZnTe layer. Under bias, electrons can hop between local charge traps with a distance of Δz and a barrier height of E_C_–E_F_ within τ_0_. As expressed in Eq. (1), it can be seen that the off current depends on the area (A) of the trap-rich region and N_T,tot_, whereas the hyperbolic sine term contributes to the electron transfer probability. The slope in the log (I)–V curve of the subthreshold regime determines Δz, where a longer Δz is associated with a lower electron hopping probability. This model also gives the relationship between V_th_ and the thickness of the chalcogenide layer. Figure S3 of the Supplementary Information show a clear linear thickness dependency of I–V responses and V_th_ in the ZnTe-based selector, thus reflecting the appreciable tunability of V_th_ for practical selection window margins. Figure 3 presents the representative I–V plots of the off current under diverse parameters necessary for analytical simulations. As shown in Fig. 3a, all devices give two different current slopes at a certain voltage boundary during voltage sweeps: linear (red line) and exponential (blue line) behaviors in currents for extremely small and subthreshold voltage regimes, respectively. The linear I–V response is explained by linear approximation of Eq. (1) with the same physical origin caused by thermal hopping. Figure 3b,c refer to the analytical simulation results for the Δz and E_C_–E_F_ levels, where the Δz of 6.5 nm is derived by extracting the slope in log (I)–V curve of the subthreshold regime. The corresponding E_C_–E_F_ level is given by adjusting the current levels from the measured off-current responses with an assistance of Δz value. The resultant value is found to be approximately 0.5 eV. To further verify the derived values of Δz and E_C_–E_F_ taken from the above analytical simulations, the temperature dependent off-current behaviors are also recorded in the range of 20–80 ℃, as shown in Fig. 3d. A high temperature leads to an increase in the off current owing to the enhanced hopping probability caused by a higher thermal energy. The resulting curves correlate well with the measurements over the entire temperature range. Such consistency implies that the conduction nature of ZnTe selectors is also similar to that observed form the previously reported OTS materials.

**Figure 4:**
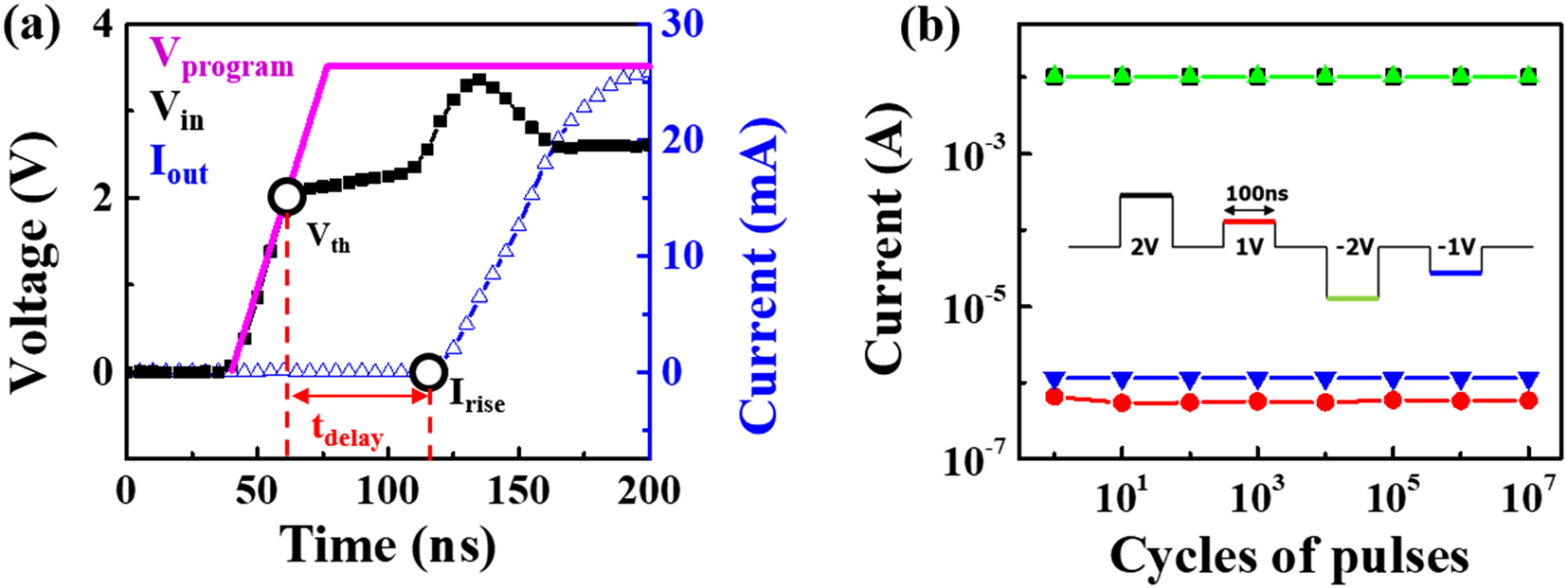
Time-resolved pulse response and endurance analyses of the ZnTe selector. (**a**) Time-resolved I–V characteristics of the ZnTe selector: programmed input voltage (purple line), actual input voltage (black line), and corresponding current (blue line) responses. The selectors stay in the off state until V > V_th_ and then the current increases after the application of V_th_ denoted as a t_delay_. (**b**) Endurance features of the selector taken by the input voltage pulse of amplitude (2 V), width (100 ns), and interval (100 ns), implying highly stable on/off ratio of both positive (black and red) and negative (green and blue) directions without exhibiting noticeable degradation up to cycles.

Figure 4 reveals the time-resolved transient pulse response and endurance tests for the ZnTe selector, where the programmed input (purple line)/output (black line) voltages and the corresponding currents (blue line) are shown. As evident in Fig. 4a, the selector remains in an off state with an extremely low current when applied voltage (V_a_) is less than V_th_. After exceeding V_th_, the current starts to increase with a delay time (t_delay_) of 60 ns and reaches the compliance current level within 40 ns. Therefore, the current rises from I_off_ to the compliance current of 25 mA within less than 100 ns, suggesting the corresponding switching speed of less than 100 ns for our device, which is closely comparable to other works. An endurance test is also conducted to test the repeatability and nonlinearity of the ZnTe selector, as shown in Fig. 4b, where a pulse measurement was done to avoid the possible disturbed current and resistance in on state induced by the incorporation of additional serial resistance. With voltages of amplitude 2 and 1 V, a width of 100 ns, and an interval of 100 ns, all devices maintained sufficient on/off currents, supporting affordable nonlinearity of 10^4^ in both positive (black and red) and negative (green and blue) directions over the repeated 10^7^ cycles.

To validate the potentials of the crossbar architecture applications, the W/ZnTe/W selector (1S) and Pt/TaO_x_/Ta ReRAM (1R) are electrically wire-connected in series, as illustrated in the Fig. S4a of the Supplementary Information. Figure S4b shows the representative I–V responses of each 1S (black line) and 1R (red line) elements, where the 1S is also serially connected with a 1 kΩ resistor (black line). The 1R device gives typical bipolar switching characteristics with an on/off ratio of about 10^2^. In an integrated 1S1R device, the nonlinearity of 1R device is greatly enhanced by the 1S, as shown in Fig. S4c. The current maintains an initially low level in the 1S device until V_th_ is reached. After exceeding V_th_, the 1S1R device switches to the on state with a lower resistance state than device 1R. This implies that the overall I–V response of 1S1R devices employs the unique advantages of each 1S and 1R device, such as high (low) on (off) current levels and an enhanced nonlinearity of 10^4^ in 1S and nonvolatile on/off ratio of 1R. Thus, we anticipate that these experimental findings can be generalized and extended to engineer future crossbar array configurations, even though more experiments are necessary in diverse multi-stacked array levels.

### Thermal stability and method for method for improvement

**Figure 5:**
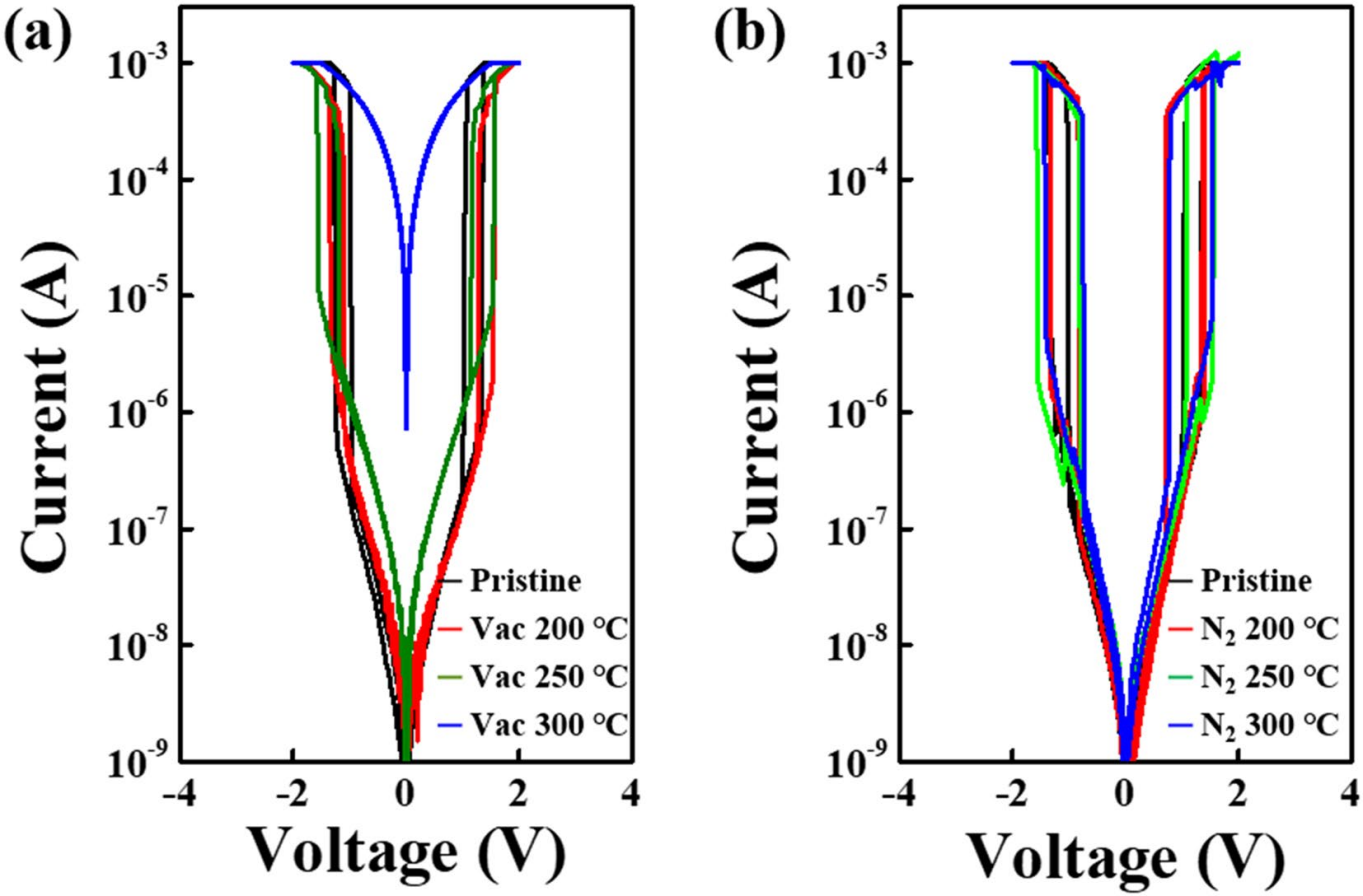
I–V characteristics annealed (**a**) in ambient condition (**b**) in a nitrogen atmosphere with different annealing temperature. I–V characteristics annealed in an air atmosphere reveals a degradation in the TS performance at temperature higher than 250 ℃. Contrastively annealed in a nitrogen ambient clearly ensuring the stable performance of TS without exhibiting no degradation up to 400 ℃.

In an effort to address the structural decomposition events observed in previously reported OTS materials upon thermal post annealing, the thermal stability features of the ZnTe selector were also tested at various temperatures. Due to the requirements of high temperature of approximately 400 ℃ at the back end of the line in 3D stacked crossbar array semiconductor processing, the temperatures ranges from 200 to 400 ℃ in vacuum and nitrogen (N_2_) atmospheres^23^. The representative I–V curves for as-grown and annealed ZnTe selectors are displayed in Fig. 5; all devices exhibit typical TS behavior, regardless of annealing conditions. As seen in the Fig. 5a, the selector annealed at 200 ℃ always maintains a relatively stable off current, whereas the selectors annealed at 250 ℃ and 300 ℃ experiences the rapid electrical degradation even at those low annealing temperatures. This deterioration effect is likely linked with the inevitable phase separation or stoichiometric decomposition of ZnTe materials upon thermal annealing. However, as seen in Fig. 5b, the selectors annealed in N_2_ atmosphere provide thermally stable features up to 400 ℃, which permits N_2_ annealing to be employed as a reliable option for achieving a thermally robust ZnTe selector. In addition, the time-resolved transient pulse response of nitrogen-annealed ZnTe exhibited similar a trend during fast transition from off state to on state without exhibiting any degradation, as evident in Fig. S5 in the Supplementary Information.

**Figure 6:**
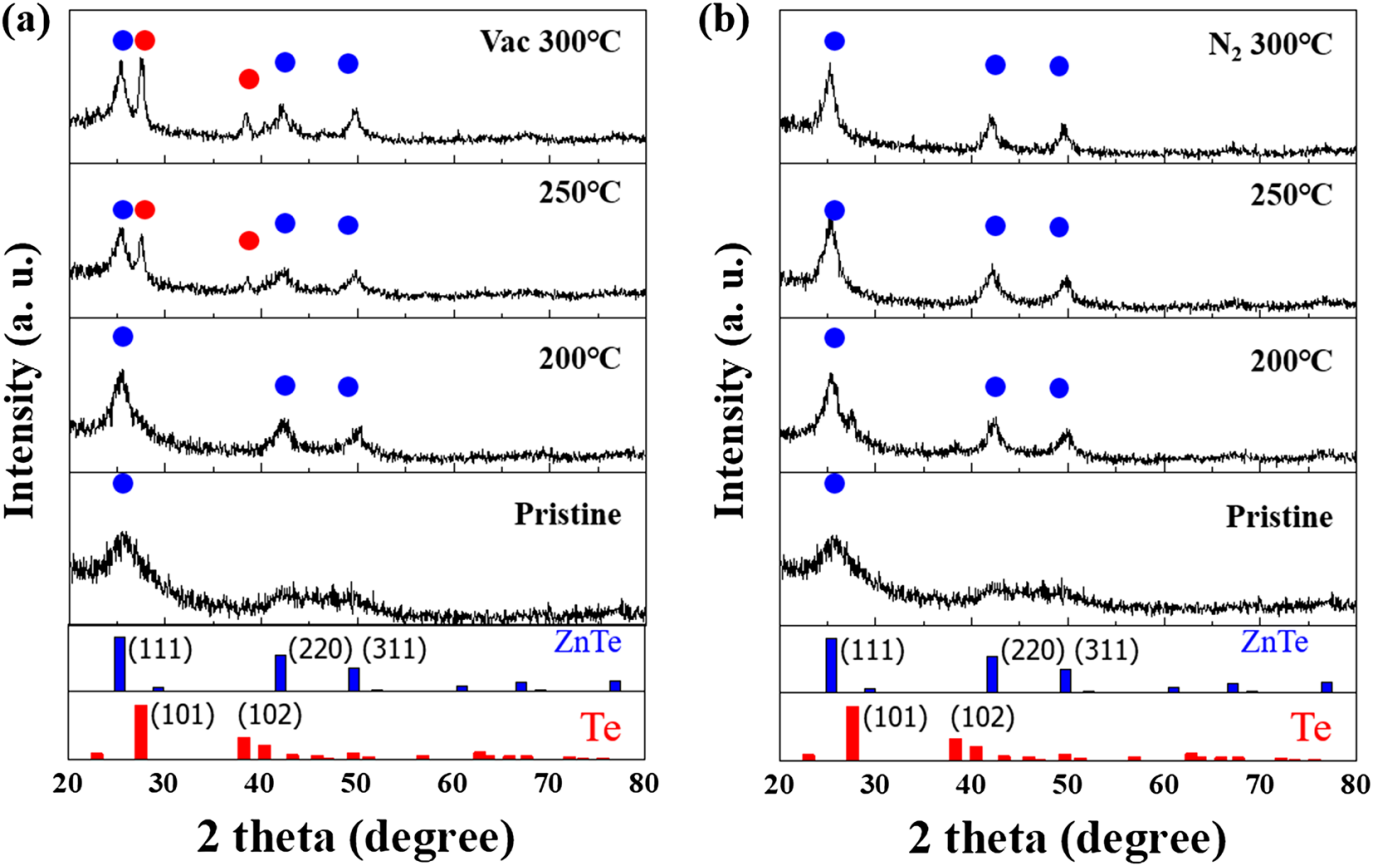
XRD patterns of ZnTe layers annealed in (**a**) vacuum and (**b**) nitrogen atmospheres. The vacuum-annealed ZnTe layer at more than 250 ℃ gives the presence of Te peaks (red), possibly arising from a phase separation event of the ZnTe layer during vacuum annealing. The presence of phase-separated Te peaks may be highly associated with the electrical degradation observed from the higher annealing temperature over 250 ℃ while the nitrogen-annealed ZnTe layer refers to thermally robust and stable patterns associated with having no phase separation characteristics.

To exploit indirect evidence for the advanced thermal stability upon N_2_ annealing, closer microstructural investigations of the ZnTe thin film were conducted. Figure 6 reveals the representative XRD patterns of ZnTe films annealed at the same temperature in vacuum and N_2_ atmospheres. As seen in Fig. 6a, the as-grown ZnTe film gives produces the expected zinc-blended FCC crystal structures. Vacuum annealing at 200 ℃ allows for the presence of slightly improved (220) and (311) peaks of ZnTe materials, while the phase separated Te peaks (red circle) are clearly shown at more than 250 ℃. This implies a possible phase-separation event in ZnTe film during vacuum annealing, which negatively affects electric performance. On the other hand, the N_2_-annealed ZnTe layer does not follow such a trend: no phase separation took place up to 400 ℃. This would explain why the N_2_-annealed device maintains the same TS behavior as as-grown selectors without having any degradation, as shown in Fig. 6b. These approximate findings only imply the eligibility of N_2_ annealing to the OTS-based selectors.

**Figure 7:**
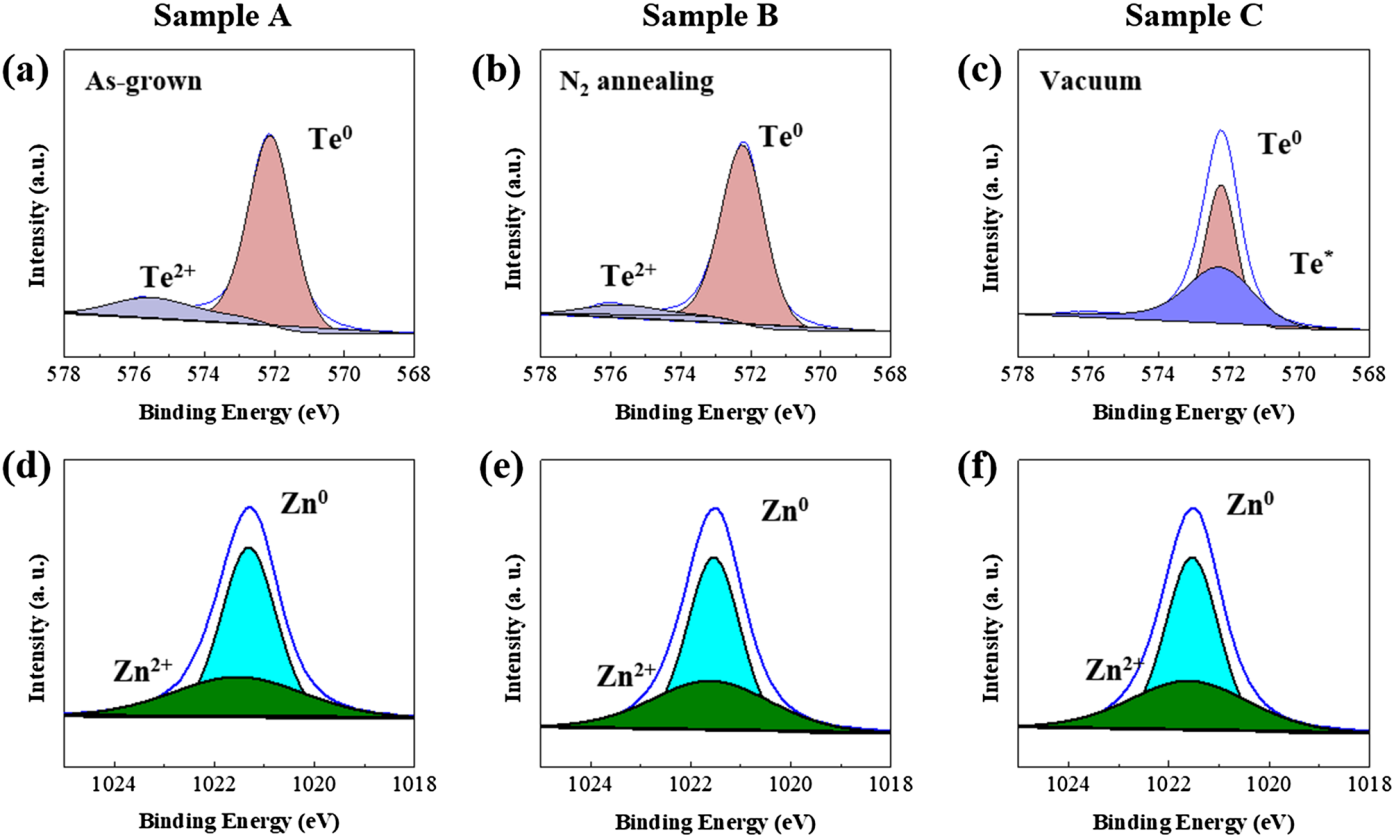
XPS analysis on pristine and annealed ZnTe layer. (**a**–**c**) XPS measurements of Te 3d_5/2_ signals. (**d**–**f**) XPS measurements of Zn 2p_3/2_ data. The proper N_2_ annealing approach maintains the pre-existing defect-related Te^2+^ peaks of Sample A while the vacuum-annealing one causes a significant variation in Te^2+^ peaks initially associated with the TS behaviors. No clear variation is detected in Zn-related peaks, which imply that Zn component is not related to the TS behaviors.

To provide further indirect evidence for the above observations regarding the effect of N_2_ annealing, XPS analyses of the as-grown (Sample A) and ZnTe layers annealed at 300 ℃ in N_2_ (Sample B) and a vacuum (Sample C) were carried out. Figure 7 shows the XPS spectra of Te 3d_5/2_ and Zn 2p_3/2_ for each sample (A–C) revealing the variation in chemical states, where each layer has the same 40 nm thickness. The Te 3d_5/2_ spectra of each sample is de-convoluted to obtain two dominant peaks at 572.11 eV and 575.54 eV, suggesting the presence of metal Te^0^ atom and Te^2+^, respectively, whereas the Zn 2p_3/2_ spectra reveal two main peaks of metal Zn^0^ atom and Zn^2+^ at 1021.31 eV and 1021.49 eV, respectively. It is widely believed that a Te chalcogenide component (Te^2+^ in this study) combines with other components. Te^2+^ initially creates the well-known lone-pair electrons, providing the localized charge traps^11,24^. Thus, suitable Te–Zn chemical binding may resemble the behavior of OTS-based selectors. However, the absence or reduction of the Te^2+^ peak from the Zn–Te binding may cause leaky I–V characteristics without exhibiting TS behavior due to a low amount of unlinked Te chains, as plotted in the blue I–V curve of Fig. 6a. Figure 7a,b shows the representative chemical binding states of Te 3d_5/2_ for Samples A and B, respectively. As seen, Sample B reveals no clear change in the Te^2+^ peak, which means that N_2_ annealing does not affect the chemical states of Te atoms in the ZnTe layer. However, vacuum annealing above 250 ℃ (Sample C) causes a variation in the binding states of Te atoms, possibly induced by the phase-separation event upon annealing^25–27^. This causes the disappearance of the Te^2+^ peak and the generation of unclear Te* elements, as evident in Fig. 7c. It is well-known that the Te atoms have various oxidation states of unknown Te* peak, which is not assigned even in other previous works^17^. However, the absence of preexisting defect-related Te^2+^ peaks observed in the as-grown sample may suggest the corresponding electrical degradation of ZnTe selectors coming from the inevitable phase-separation event upon vacuum annealing. Figure 7d–f reveals Zn peaks with no clear peak variation in each sample, regardless of annealing conditions. This suggests that the Zn component has no significant impact on the electrical TS performance. Our observations from the XRD and XPS measurements suggest that a suitable amount of Te^2+^ in ZnTe active layer may be essential to the TS observation, even though more results and comparisons are needed to establish a clearer explanation for the origin of the electrical degradation upon annealing.

## Conclusion

In this study, we address the performance and thermal stability of simple binary ZnTe selectors. Diverse electrical parameters in off current regimes were mainly determined by applying the Poole–Frankel conduction model to experimental observations, yielding information on the generation of local CFs and OTS behaviors. Simple structural analyses revealed that the representative electrical degradation upon vacuum annealing may be ascribed to a phase-separation event of ZnTe layer, while a simple N_2_-annealing approach contributes to the presence of thermally robust stability of the ZnTe selector without having a phase-separation event, even at higher annealing temperatures. We anticipate that the above experimental findings will lead to future practical applications for 3D stackable/scalable crossbar array frames.

## Method

### Sample fabrication

The micro-and nano-sized W bottom electrodes are, at first, prepared on the Si_3_N_4_ substrates to define the device cell areas during electrical analyses and then followed by the deposition of 40-nm-thick Zn_0.5_Te_0.5_ (ZnTe) films through a radio frequency (RF) magnetron sputtering using a single ZnTe target of the 1:1 composition ratio. The base and working pressures for the growth of ZnTe thin films are 2 × 10^–7^ Torr and 3 × 10^–3^ Torr under Ar only ambient, respectively. The top W electrode dimension is a 50 μm square pattern defined by a typical photolithography approach. Two post annealing processes are also conducted in vacuum and nitrogen ambient of 10^–2^ Torr and 2 Torr for comparison, respectively.

### Chemical and electrical characterization

Crystal structure and chemical bonding analyses of ZnTe films verse annealing temperatures are taken by the high-resolution X-ray diffractometer (XRD) and the X-ray photoemission spectroscopy (XPS) using a K-Alpha+. DC current–voltage (I–V) and pulse characteristics are determined using a Keithley 4200 semiconductor parameter analyzer (Keithley 4200 SPA, Keithley Instruments, Inc.), where the compliance current of 1 mA is set to prevent the problem of possible breakdown of ZnTe selectors.

## Supplementary information

Supplementary Figures.
