# Supplementary material for: Bidirectional-nonlinear threshold switching behaviors and thermally robust stability of ZnTe selectors by nitrogen annealing
Source: Sci Rep. 2020 Oct 1;10:16286. doi: 10.1038/s41598-020-73407-3 (PMC7529746; doi:10.1038/s41598-020-73407-3)
Supplement: Supplementary file 1 — Supplementary Figures. [file 41598_2020_73407_MOESM1_ESM.docx]

SUPPLEMENTARY INFORMATION

Correspondence and requests for materials should be addressed to J.H (jphong@hanyang.ac.kr) and JY. Yang (jungyup.yang@kunsan.ac.kr)

Bidirectional-nonlinear threshold switching behaviors and thermally robust stability of ZnTe selectors by nitrogen annealing

Gabriel Jang^1, †^, Mihyun Park^2,†^, Da Seul Hyeon^1^, Woojong Kim^2^, JungYup Yang^3,^*, and Jinpyo Hong^1,2,^*

^1^Research Institute of Natural Science, Novel Functional Materials and Device Laboratory, Department of Physics, Hanyang University, Seoul 04763, Republic of Korea

^2^Division of Nano-Scale Semiconductor Engineering, Hanyang University, Seoul 04763, Republic of Korea

^3^Department of Physics, Kunsan National University, Gunsan 54150, Republic of Korea

* Corresponding authors

^†^ These authors contributed equally to this work**I-V response under current and voltage sweep mode.**

**
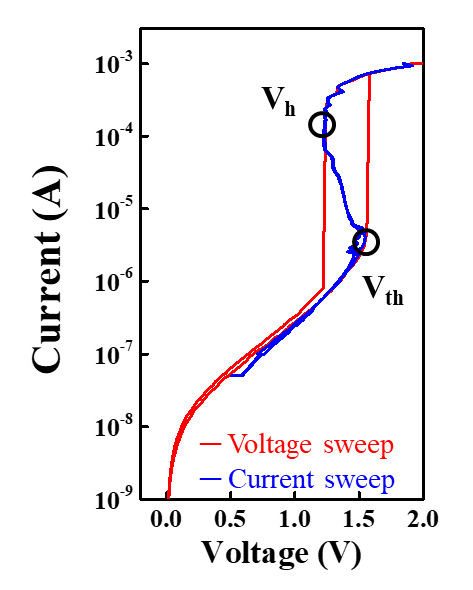
**

**Figure. S1** Comparison of TS curves under voltage (red line) and current (blue line) sweep modes. The TS curves under voltage sweep mode displays the typical abrupt current increase and decrease at the threshold (V_th_) and hold-on (V_h_) voltages, respectively, while the TS curve in current sweep mode is an S-shaped curve ensuring a negative differential resistance feature.

1. **Consistent threshold and holding voltage of the selector connected with various series resistor.**


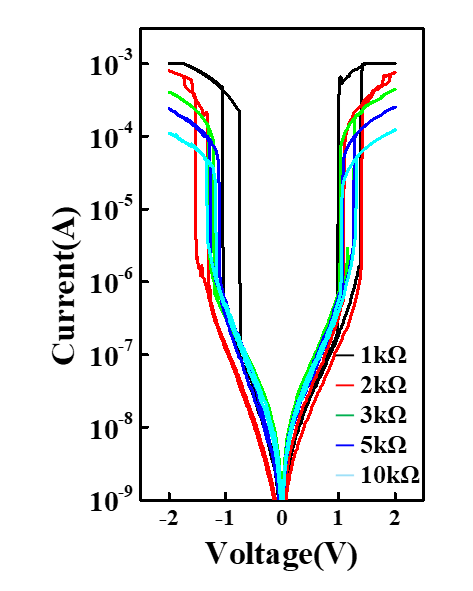


**Figure S2.** Serially connected external resistance dependence of the ZnTe selector. There is no significant variation in the operation voltage, V_th_ and V_h_, except for the off- and on-current level. The reason why V_th_ does not change even under various magnitudes of the external resistance is due to the fact that the resistance of the selector is still higher than the connected resistance of the external resistor.

1. **Serially connected external resistance dependence of the ZnTe selector.**


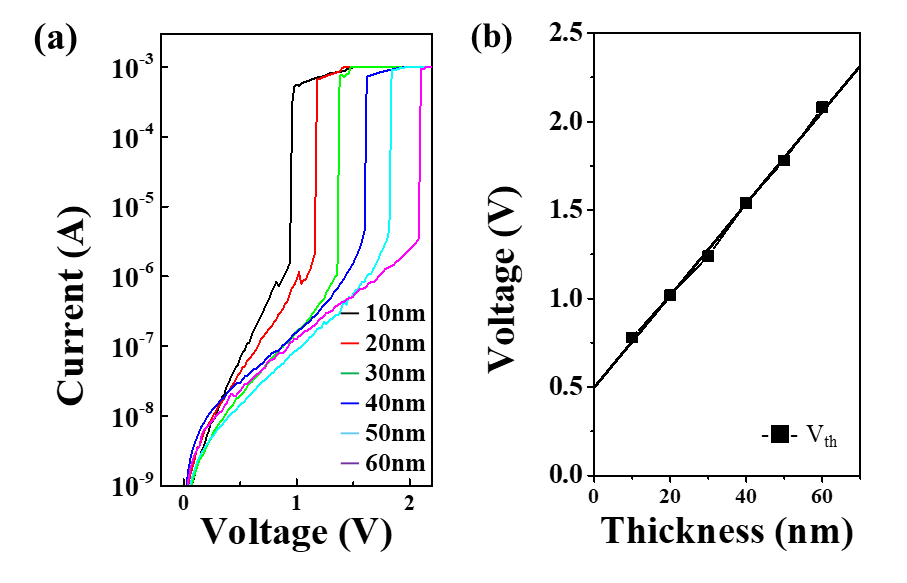


**Figure. S3.** Thickness-dependent TS responses of the ZnTe selector. a) Typical I-V features and b) Linear behavior of V_th_ with increasing ZnTe layer thicknesses. This dependence indicates that TS switching can be triggered by simply adjusting the thickness of the active layer.

1. **Serially connected external resistance dependence of the ZnTe selector.**


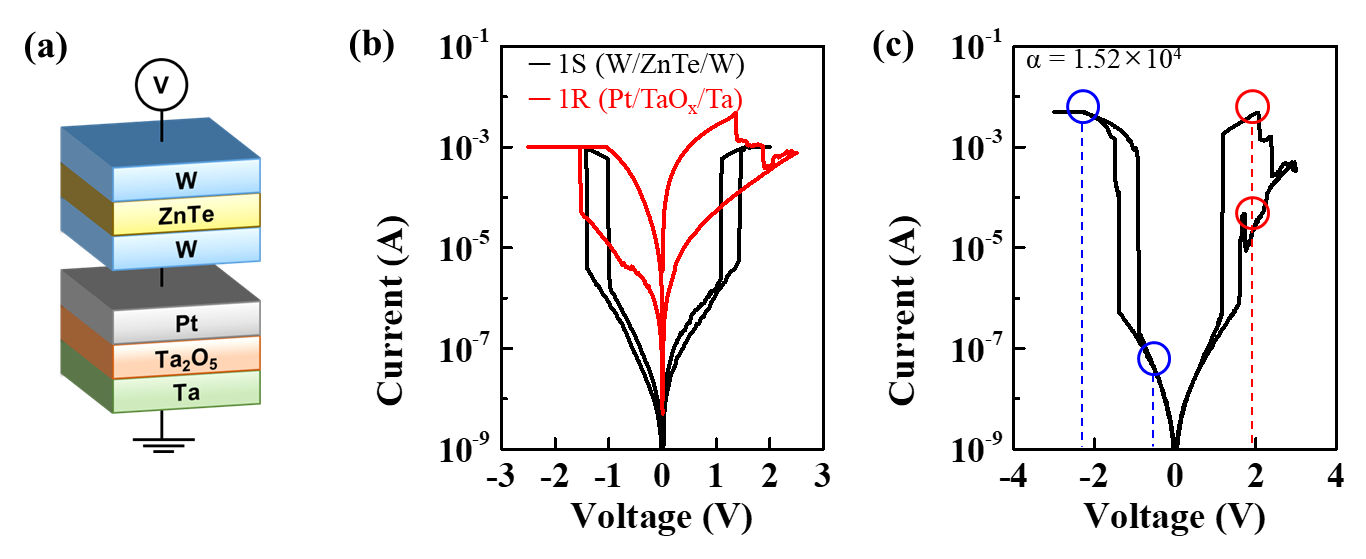


**Figure S4.** Electrical characteristics of 1S1R frame. a) Schematic diagram of a serially connected selector (1S) and resistive memory (1R) through the electrical wire connection. b) Representative I-V responses of the 1S (black line) and 1R (red line) elements. c) Representative I-V features of 1S1R frame, clearly providing a large memory window and low off-current levels.

1. **Time-resolved pulse measurement of ZnTe selectors**


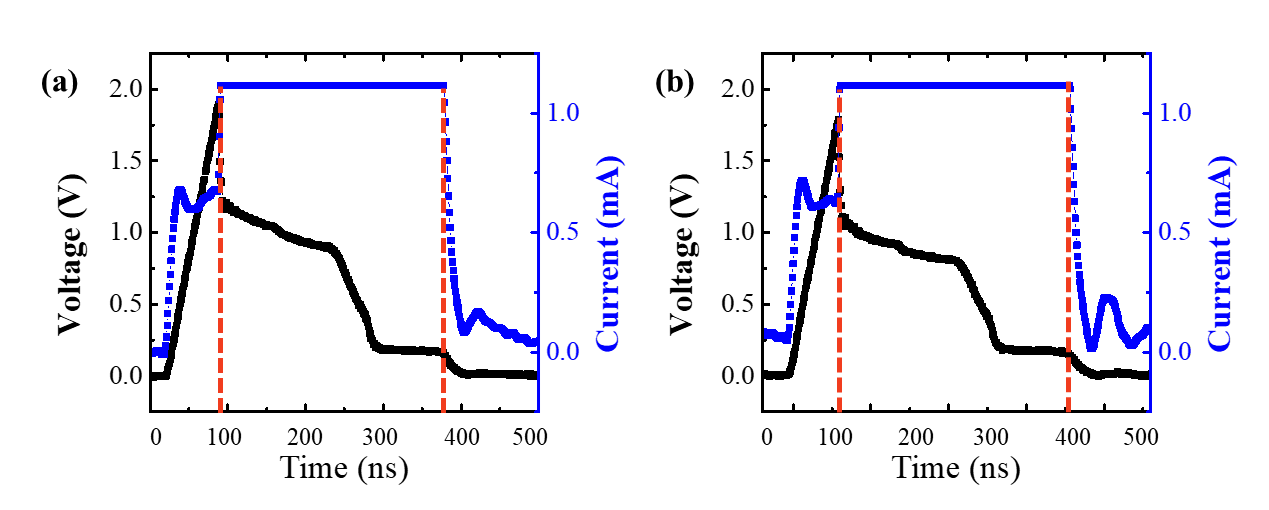


**Figure S5.** Time-resolved pulse measurement of ZnTe selector a) in pristine and b) annealed at 400 ℃ in nitrogen ambient, which allows for no clear degradation in electrical pulse performance.
